# Supplementary material for: Long-term outcomes of survivors of neonatal insults: A systematic review and meta-analysis
Source: PLoS One. 2020 Apr 24;15(4):e0231947. doi: 10.1371/journal.pone.0231947 (PMC7182387; doi:10.1371/journal.pone.0231947)
Supplement: S1 Appendix — (DOCX) [file pone.0231947.s002.docx]

Jaundice OR Hyperbilirubinemia OR Sepsis OR Birth asphyxia OR Hypoxic-ischemic encephalopathy OR Preterm birth OR Tetanus OR Meningitis, OR Herpes OR Rubella or Cytomegalovirus OR Syphilis, OR HIV or Malaria AND Neonatal OR Congenital OR intrauterine AND Deficit OR Sequela OR Effect OR Consequence OR Disability OR Impairment AND Neurologic OR Neurocognitive OR Neurobehavioral Manifestations OR Behavioral problems OR mental problems OR Neuromuscular OR Neurosensory OR Pain OR Dyskinesia OR Meningism OR Paralysis OR Seizures OR Sensation disorder”
