# Supplementary material for: Long-term outcomes of survivors of neonatal insults: A systematic review and meta-analysis
Source: PLoS One. 2020 Apr 24;15(4):e0231947. doi: 10.1371/journal.pone.0231947 (PMC7182387; doi:10.1371/journal.pone.0231947)
Supplement: S2 Appendix — (DOCX) [file pone.0231947.s003.docx]

**Included Studies**

1. Anderson PJ, Treyvaud K, Neil JJ, Cheong JL, Hunt RW, Thompson DK, et al. Associations of newborn brain magnetic resonance imaging with long-term neurodevelopmental impairments in very preterm children. The Journal of Pediatrics. 2017;187:58-65. e1.

2. Andrews WW, Cliver SP, Biasini F, Peralta-Carcelen AM, Rector R, Alriksson-Schmidt AI, et al. Early preterm birth: association between in utero exposure to acute inflammation and severe neurodevelopmental disability at 6 years of age. American Journal of Obstetrics and Gynecology. 2008;198(4):466. e1-. e11.

3. Anvar B, Mencher GT, Keet SJ. Hearing loss and congenital rubella in Atlantic Canada. Ear and Hearing. 1984;5(6):340-5.

4. Barnett A, Mercuri E, Rutherford M, Haataja L, Frisone M, Henderson S, et al. Neurological and perceptual-motor outcome at 5-6 years of age in children with neonatal encephalopathy: relationship with neonatal brain MRI. Neuropediatrics. 2002;33(05):242-8.

5. Beukers F, Aarnoudse-Moens CS, van Weissenbruch MM, Ganzevoort W, van Goudoever JB, van Wassenaer-Leemhuis AG. Fetal growth restriction with brain sparing: neurocognitive and behavioral outcomes at 12 years of age. The Journal of Pediatrics. 2017;188:103-9. e2.

6. Bora S, Pritchard VE, Moor S, Austin NC, Woodward LJ. Emotional and behavioural adjustment of children born very preterm at early school age. Journal of Paediatrics and Child Health. 2011;47(12):863-9.

7. Bos AF, Roze E. Neurodevelopmental outcome in preterm infants. Developmental Medicine & Child Neurology. 2011;53:35-9.

8. Boskabadi H, Maamouri G, Mafinejad S, Rezagholizadeh F. Clinical course and prognosis of hemolytic jaundice in neonates in North East of Iran. Macedonian Journal of Medical Sciences. 2011;4(4):403-7.

9. Brévaut-Malaty V, Busuttil M, Einaudi M-A, Monnier A-S, D’Ercole C, Gire C. Longitudinal follow-up of a cohort of 350 singleton infants born at less than 32 weeks of amenorrhea: neurocognitive screening, academic outcome, and perinatal factors. European Journal of Obstetrics & Gynecology and Reproductive Biology. 2010;150(1):13-8.

10. Chen M-H, Su T-P, Chen Y-S, Hsu J-W, Huang K-L, Chang W-H, et al. Is neonatal jaundice associated with autism spectrum disorder, attention deficit hyperactivity disorder, and other psychological development? A nationwide prospective study. Research in Autism Spectrum Disorders. 2014;8(6):625-32.

11. Chess S, Fernandez P, Korn S. Behavioral consequences of congenital rubella. The Journal of Pediatrics. 1978;93(4):699-703.

12. Culley P, Powell J, Waterhouse J, Wood B. Sequelae of neonatal jaundice. Br Med J. 1970;3(5719):383-6.

13. Desmond MM, Fisher ES, Vorderman AL, Schaffer HG, Andrew LP, Zion TE, et al. The longitudinal course of congenital rubellaencephalitis in nonretarded children. The Journal of Pediatrics. 1978;93(4):584-91.

14. Dotinga BM, de Winter AF, Bocca-Tjeertes IF, Kerstjens JM, Reijneveld SA, Bos AF. Longitudinal growth and emotional and behavioral problems at age 7 in moderate and late preterms. PLOS ONE. 2019;14(1):e0211427.

15. Easson K, Dahan-Oliel N, Rohlicek C, Sahakian S, Brossard-Racine M, Mazer B, et al. a comparison of developmental outcomes of adolescent neonatal intensive care unit survivors born with a congenital heart defect or born preterm. The Journal of Pediatrics. 2019;207:34-41. e2.

16. Foulder-Hughes L, Cooke R. Motor, cognitive, and behavioural disorders in children born very preterm. Developmental medicine and child neurology. 2003;45(2):97-103.

17. Geldof CJ, van Hus JW, Jeukens-Visser M, Nollet F, Kok JH, Oosterlaan J, et al. Deficits in vision and visual attention associated with motor performance of very preterm/very low birth weight children. Research in Developmental Disabilities. 2016;53:258-66.

18. Geldof CJ, van Wassenaer-Leemhuis AG, Dik M, Kok JH, Oosterlaan J. A functional approach to cerebral visual impairments in very preterm/very-low-birth-weight children. Pediatric Research. 2015;78(2):190.

19. Guellec I, Lapillonne A, Marret S, Picaud J-C, Mitanchez D, Charkaluk M-L, et al. Effect of intra-and extrauterine growth on long-term neurologic outcomes of very preterm infants. The Journal of Pediatrics. 2016;175:93-9. e1.

20. Guellec I, Lapillonne A, Renolleau S, Charlaluk M-L, Roze J-C, Marret S, et al. Neurologic outcomes at school age in very preterm infants born with severe or mild growth restriction. Pediatrics. 2011;127(4):e883-e91.

21. Hadders-Algra M, Huisjes H, Touwen B. Preterm or small-for-gestational-age infants. European Jurnal of Pediatrics. 1988;147(5):460-7.

22. Heinonen K, Eriksson JG, Lahti J, Kajantie E, Pesonen A-K, Tuovinen S, et al. Late preterm birth and neurocognitive performance in late adulthood: a birth cohort study. Pediatrics. 2015;135(4):e818-e25.

23. Hirvonen M, Ojala R, Korhonen P, Haataja P, Eriksson K, Rantanen K, et al. Intellectual disability in children aged less than seven years born moderately and late preterm compared with very preterm and term‐born children–a nationwide birth cohort study. Journal of Intellectual Disability Research. 2017;61(11):1034-54.

24. Hokkanen L, Launes J, Michelsson K. Adult neurobehavioral outcome of hyperbilirubinemia in full term neonates—a 30 year prospective follow-up study. PeerJ. 2014;2:e294.

25. Holmström G, Larsson E. Long-term follow-up of visual functions in prematurely born children—a prospective population-based study up to 10 years of age. Journal of American Association for Pediatric Ophthalmology and Strabismus. 2008;12(2):157-62.

26. Huddy C, Johnson A, Hope P. Educational and behavioural problems in babies of 32–35 weeks gestation. Archives of Disease in Childhood-Fetal and Neonatal Edition. 2001;85(1):F23-F8.

27. Ishikawa T, Ogawa Y, Kanayama M, Wada Y. Long-term prognosis of asphyxiated full-term neonates with CNS complications. Brain and Development. 1987;9(1):48-53.

28. Jurgens-Van der Zee A, Bierman-van Eendenburg M, Fidler V, Olinga A, Visch J, Touwen B, et al. Preterm birth, growth retardation and acidemia in relation to neurological abnormality of the newborn. Early Human Development. 1979;3(2):141-54.

29. Koç Ö, Kavuncuoğlu S, Ramoğlu MG, Aldemir E, Aktalay A, Eras Z. School performance and neurodevelopment of very low birth weight preterm infants: first report from Turkey. Journal of Child Neurology. 2016;31(2):170-6.

30. Korndewal MJ, Oudesluys‐Murphy AM, Kroes AC, Van der Sande MA, De Melker HE, Vossen AC. Long‐term impairment attributable to congenital cytomegalovirus infection: a retrospective cohort study. Developmental Medicine & Child Neurology. 2017;59(12):1261-8.

31. Kuban KC, Joseph RM, O'shea TM, Allred EN, Heeren T, Douglass L, et al. Girls and boys born before 28 weeks gestation: risks of cognitive, behavioral, and neurologic outcomes at age 10 years. The Journal of Pediatrics. 2016;173:69-75. e1.

32. Kuzniewicz M, Newman TB. Interaction of hemolysis and hyperbilirubinemia on neurodevelopmental outcomes in the collaborative perinatal project. Pediatrics. 2009;123(3):1045-50.

33. Lacey JL, PhD DJHSM. Assessment of preterm infants in the intensive‐care unit to predict cerebral palsy and motor outcome at 6 years. Developmental Medicine & Child Neurology. 1998;40(5):310-8.

34. Lanzieri TM, Chung W, Flores M, Blum P, Caviness AC, Bialek SR, et al. Hearing loss in children with asymptomatic congenital cytomegalovirus infection. Pediatrics. 2017;139(3):e20162610.

35. Leitner Y, Fattal-Valevski A, Geva R, Eshel R, Toledano-Alhadef H, Rotstein M, et al. Neurodevelopmental outcome of children with intrauterine growth retardation: a longitudinal, 10-year prospective study. Journal of Child Neurology. 2007;22(5):580-7.

36. Lindström K, Lindblad F, Hjern A. Preterm birth and attention-deficit/hyperactivity disorder in schoolchildren. Pediatrics. 2011;127(5):858-65.

37. Luu TM, Ment LR, Schneider KC, Katz KH, Allan WC, Vohr BR. Lasting effects of preterm birth and neonatal brain hemorrhage at 12 years of age. Pediatrics. 2009;123(3):1037-44.

38. Marlow N, Rose A, Rands C, Draper E. Neuropsychological and educational problems at school age associated with neonatal encephalopathy. Archives of Disease in Childhood-Fetal and Neonatal Edition. 2005;90(5):F380-F7.

39. McIntosh E, Menser M. A fifty-year follow-up of congenital rubella. The Lancet. 1992;340(8816):414-5.

40. Menser M, Dods L, Harley J. A twenty-five-year follow-up of congenital rubella. The Lancet. 1967;290(7530):1347-50.

41. Natarajan G, Shankaran S, Pappas A, Bann C, Tyson JE, McDonald S, et al. Functional status at 18 months of age as a predictor of childhood disability after neonatal hypoxic‐ischemic encephalopathy. Developmental Medicine & Child Neurology. 2014;56(11):1052-8.

42. Newman TB, Klebanoff MA. Neonatal hyperbilirubinemia and long-term outcome: another look at the Collaborative Perinatal Project. Pediatrics. 1993;92(5):651-7.

43. Pappas A, Shankaran S, McDonald SA, Vohr BR, Hintz SR, Ehrenkranz RA, et al. Cognitive outcomes after neonatal encephalopathy. Pediatrics. 2015;135(3):e624-e34.

44. Seidman DS, Paz I, Stevenson DK, Laor A, Danon YL, Gale R. Neonatal hyperbilirubinemia and physical and cognitive performance at 17 years of age. Pediatrics. 1991;88(4):828-33.

45. Stevens J, Eames M, Kent A, Halket S, Holt D, Harvey D. Long term outcome of neonatal meningitis. Archives of Disease in Childhood-Fetal and Neonatal Edition. 2003;88(3):F179-F84.

46. Talge NM, Holzman C, Wang J, Lucia V, Gardiner J, Breslau N. Late-preterm birth and its association with cognitive and socioemotional outcomes at 6 years of age. Pediatrics. 2010;126(6):1124-31.

47. Van Baar AL, Van Wassenaer AG, Briët JM, Dekker FW, Kok JH. Very preterm birth is associated with disabilities in multiple developmental domains. Journal of Pediatric Psychology. 2005;30(3):247-55.

48. van Handel M, Swaab H, de Vries LS, Jongmans MJ. Behavioral outcome in children with a history of neonatal encephalopathy following perinatal asphyxia. Journal of Pediatric Psychology. 2009;35(3):286-95.

49. van Kooij BJ, van Handel M, Nievelstein RA, Groenendaal F, Jongmans MJ, de Vries LS. Serial MRI and neurodevelopmental outcome in 9-to 10-year-old children with neonatal encephalopathy. The Journal of Pediatrics. 2010;157(2):221-7. e2.

50. van Schie PE, Schijns J, Becher JG, Barkhof F, van Weissenbruch MM, Vermeulen RJ. Long-term motor and behavioral outcome after perinatal hypoxic-ischemic encephalopathy. European Journal of Paediatric Neurology. 2015;19(3):354-9.

51. Vandborg PK, Hansen BM, Greisen G, Mathiasen R, Kasper F, Ebbesen F. Follow‐up of extreme neonatal hyperbilirubinaemia in 5‐to 10‐year‐old children: a Danish population‐based study. Developmental Medicine & Child Neurology. 2015;57(4):378-84.

52. Zhang X-W, Li F, Yu X-W, Shi X-W, Shi J, Zhang J-P. Physical and intellectual development in children with asymptomatic congenital cytomegalovirus infection: a longitudinal cohort study in Qinba mountain area, China. Journal of Clinical Virology. 2007;40(3):180-5.
