# Supplementary material for: Long-term outcomes of survivors of neonatal insults: A systematic review and meta-analysis
Source: PLoS One. 2020 Apr 24;15(4):e0231947. doi: 10.1371/journal.pone.0231947 (PMC7182387; doi:10.1371/journal.pone.0231947)
Supplement: S1 Table — (DOCX) [file pone.0231947.s004.docx]

Table S1

Characteristics of Studies Included in the Review

| Author | Sample size | Age at Follow-up (in years) | Country | Type of study | Neonatal Insult | Definition of the Neonatal Insult | Assessment Tool | Outcome | Results |
| --- | --- | --- | --- | --- | --- | --- | --- | --- | --- |
| (Stevens et al., 2003) | 273 (exposed= 111) | 9 to 10 | Canada | Prospective | Bacterial Meningitis | Positive cerebrospinal fluid culture | Wechsler Intelligence Scale for Children, movement assessment battery Sonksen-Silver acuity system, audiometer | Intellectual ability, motor, visual, and hearing function | 3.6% had SNHL, 2.7% had persisting hydrocephalus 5.4% of cases and 1.7% of  controls had treatment for seizures |
| (Ishikawa, Ogawa, Kanayama, & Wada, 1987) | 54 | 10 | Japan | Prospective | Birth asphyxia |  | Tanaka-Binet Intellectual Test or Wechsler Intelligence Scale for Children, Tsumori-Inage scales of psychomotor for children | Intellectual, neurological, and motor functions | 24% (13) of 55 children showed major disability |
| (Korndewal et al., 2017) | 407 (exposed =113) | 6 | Netherlands | Retrospective | Congenital cytomegalovirus infection | CMV was diagnosed retrospectively in stored neonatal dried blood spots using polymerase chain reactions |  | SNHL, cognitive, speech, language development. | SNHL and cognitive, motor, and speech-language impairment, was seen in 24.8% cases compared with 12.0% of controls |
| (Lanzieri et al., 2017) | 143 (exposed=92) | 10 to 18 | USA | Prospective | Congenital cytomegalovirus infection | were screened for congenital CMV infection via urine culture collected within 3 days of life | Auditory brainstem response | SNHL | 10, 14, and 18 years; 15,19, and 20 had hearing loss of the cases respectively. 10,14, and 18 years; 5,8,8 had hearing loss respectively for controls |
| (Zhang et al., 2007) | 99 (exposed = 49) | 6 | China | Prospective | Congenital cytomegalovirus infection | Congenital CMV infection was diagnosed if a specific fragment of HCMV-DNA was found by means of PCR | Head circumference, length and weight and Gesell Developmental Schedule | Physical and intellectual development outcomes | The intellectual development was disproportion in cases compared to controls |
| (Anvar, Mencher, & Keet, 1984) | 41 | 7 to 9 | Canada | Retrospective | Congenital Rubella |  | Auditory brainstem response | Hearing loss | Most children had normal Type A tympanogram  6 had type AD and/or type B tympanograms. |
| (Chess, Fernandez, & Korn, 1978) | 205 | 8 to 9 | USA | Prospective | Congenital Rubella |  |  | Behavioral function | at preschool 37% were retarded 15% had reactive behavior disorder and 7% had atttism. |
| (Desmond et al., 1978) | 29 | 9 to 12 | USA | Prospective | Congenital Rubella |  | Neurologic examination, physical examination, otolaryngologic, audio, and opthalmic evaluations, mental testing, and parental interviews | Neurologic function and intellectual function | 69% had motor problems  66% behavioral disturbances |
| (McIntosh & Menser, 1992) | 40 | 50 | UK | Retrospective | Congenital Rubella |  | Medical review | Neurological ability | All had hearing impairing 3-eye defects 2 -mentally retarded 3 -small mental handicap |
| (Menser et al., 1967) | 50 | 25 | UK | Retrospective | Congenital Rubella |  | Medical review |  | 48 deaf 26 typical cataracts 5 below the 10th percentile for- weight-height 11 had congenital cardiovascular defects 3 had systemic arterial hypertension 1 undiagnosed diabetes mellitus 5 were mentally defective |
| (Beukers et al., 2017) | 128 (exposed=96) | 12 | Netherlands | Prospective | Fetal Growth Restriction | BWR <10th percentile was defined as growth restricted | Wechsler Intelligence Scale for Children, Amsterdam Neuropsychological Tasks; Child Behavior Checklist | Neurocognitive outcomes | Parents of children with FGR reported more social problems |
| (Guellec et al., 2011) | 1364 (exposed=536) | 8 | France | Prospective | Fetal Growth Restriction | classic SGA (<10th percentile) and also a category called mild- SGA (10th–19th centile) | Kaufman Assessment Battery for Children, Mental Processing Composite scale, Strength and Difficulties Questionnaire | Neurocognitive outcomes | SGA children had minor cognitive difficulties, inattention-hyperactivity symptoms, and school difficulties compared with AGA children. |
| (Guellec et al., 2016) | 1305 | 8 | France | Prospective | Fetal Growth Restriction | small for gestational-age (SGA) by birth weight below 2 SD. | medical examination, Kauffman Assessment Battery for Children, Strengths and Difficulties questionnaire | Intellectual ability and behavior and emotional problems | Poor neurologic outcome for AGA and SGA preterm infants. SGA infants showed behavioral problems and cognitive deficiency |
| (Leitner et al., 2007) | 186 (exposed=123) | 9 to 10 | Israel | Prospective | Fetal Growth Restriction |  | Wechsler Intelligence Scale for Children, Kauffman Assessment Battery for Children, | Neurodevelopmental | Lower neurodevelopmental scores for children with IUGR compared to controls |
| (Barnett et al., 2002) | 46 (exposed=22) | 5.5 to 6.5 | UK | Prospective | HIE | Apgar scores of 5 or below at 1 minute | Movement Assessment Battery for Children, Wechsler Pre-School and Primary Scale of Intelligence | neurocognitive outcomes | 15%-minor neurological dysfunction,  2 %- cognitive impairment  47% were normal. |
| (Lindström, Lindblad, & Hjern, 2011b) | 43 | 15 to 19 | Sweden | Prospective | HIE | Apgar score <7 at 5 minutes | Assessed by a neonatologist and a neurologist | neurocognitive outcomes | 51% had cognitive dysfunctions without CP  19% had no obvious impairments |
| (Marlow et al., 2005) | 65 | 7 to 9 | UK | Retrospective | HIE |  | British ability scales school age battery 6 and NEPSY,Griffiths developmental scale | Cognition function |  |
| (Natarajan et al., 2014) | 111 | 6 to 7 | USA | Prospective | HIE |  | Gross Motor Function Classification System Cerebral palsy was classified on the basis of the Surveillance of Cerebral Palsy in Europe, Wechsler Preschool and Primary Scale of Intelligence III | neurodevelopmental outcomes, Cerebral palsy, moto | 33.3% had severe disability |
| (Pappas et al., 2015) | 110 | 6 to 7 | USA | Prospective | HIE |  | Bayley Scales of Infant Development, Wechsler intelligence scales, NEPSY Developmental Neuropsychological Assessment, GMFCS | IQ, neurodevelopmental function, motor function, education achievement | 30%- special education services, 7-9% behavior problems, 96% with CP had cognitive impairment, |
| (van Handel et al., 2009) | 134 (exposed=47) | 9 to 10 | Netherlands | Prospective | HIE | (1) signs of fetal distress (2) Apgar score below seven at 5min, (3) arterial umbilical pH below 7.10, (4) delay in onset of spontaneous respiration, (5) multiorgan failure | CBCL, TRF, DSM, Children’s Social Behavior Questionnaire | Behavior problems, anxiety disorders, mood disorders, disruptive disorders | Cases showed more problematic behaviors than controls, |
| (van Kooij et al., 2010) | 128 (exposed=77) | 9 to 10 | Netherlands | Prospective | HIE |  | MRI, Wechsler Intelligence Scale for Children, GMFCS | Motor, intelligence, |  |
| (Boskabadi, Maamouri, Mafinejad, & Rezagholizadeh, 2011) | 759 | 7 | Iran | Prospective | Neonatal Jaundice | Clinical jaundice is diagnosed by yellowish color of sclera, mucosal and skin. | Clinical evaluation | Neurological functioning | prevalence rate of kernicterus was 1.5 in cases |
| (Chen et al., 2014a) | 10080 (exposed=2016) | 6 | Taiwan | Prospective | Neonatal Jaundice | ICD-9-CM codes: 774 |  | Intellectual ability | 2.5% of the cases and 111(1.5%) of the controls had intellectual disability |
| (Culley, Powell, Waterhouse, & Wood, 1970) | 371 | 6 | UK | Prospective | Neonatal Jaundice |  | Stanford Binet Intelligence scale | Intellectual ability | No relationship between depth of jaundice and IQ |
| (Hokkanen et al., 2014) | 210 | 30 | Finland | Prospective | Neonatal Jaundice | bilirubin concentrations > 340 μmol/l or required blood exchange transfusion | reported psychiatric problems, DHD Current Symptoms Scale as well as the ADHD Childhood Symptoms Scale, | behavioral outcome, cognitive outcome, Educational outcome, Occupational achievement, Social functioning, Life satisfaction | 45% HB group were affected by cognitive abnormalities in childhood and continued to experience problems in adulthood |
| (Kuzniewicz & Newman, 2009) | 32808 | 7 to 8 | USA | Prospective | Neonatal Jaundice |  | Wechsler Intelligence Scale for Children, pure tone audiometer | cognitive function, hearing ability | among infants with a TSB level of ≥25 mg/dL, those with a positive direct antiglobulin test (DAT) had significantly lower IQs compared with infants with a negative DAT |
| (Newman & Klebanoff, 1993) | 41324 | 7 | USA | Prospective | Neonatal jaundice | Bilirubin > 171 m/l. Bilirubin measured using diazo method and spectrophotometry | Weschsler Intelligence Scale, Neurological examination, pure tone audiometry | IQ, neurological function, hearing function | Hearing loss and abnormal neurological function |
| (Seidman et al., 1991) | 1948 | 17 | Israel | Prospective | Neonatal Jaundice | moderate NNJ was defined as serum bilirubin levels of 86 to 137 micro mols/l, severe bilirubinemia was defined as serum bilirubin level exceed exceeding 256 micro mols/l | Wechsler Adult Intelligence Scale | Intellectual ability | the mean IQ score was significantly lower for the male subjects that were found to have severe NNJ |
| (Vandborg et al., 2015) | 330 | 5 to 10 | Denmark | Prospective | Neonatal Jaundice | TSB ≥450 lmol/L (26.3mg/dL) | Movement Assessment Battery for Children II, Madsen Micromate 304 screening audiometer, BRIEF | motor function, behavior funcion, hearing function | No children had significant impairment |
| (Anderson et al., 2017) | 187 | 7 | Australia | Prospective | Preterm birth | (<30 weeks of gestation or <1250 g) | MRI, Wechsler Abbreviated Scale of Intelligence, Wide Range Achievement Test-4, Movement Assessment Battery for Children-Second Edition, Strengths and Difficulties Questionnaire (SDQ) | neurological function, intelligence, neurocognitive | White and grey matter abnormality scores were related to poorer IQ, spelling, math computation and motor function. |
| (Andrews et al., 2008) | 261 | 6 to 8 | UK | Prospective | Preterm birth | 23 and <32 weeks | Wechsler Intelligence Scale for Children-IV, Pea- body Picture Vocabulary Test, | Intellectual ability and neurologic function | Children with an IQ < 70, cerebral palsy, and a major disability at age 6 years had significantly lower mean delivery gestational ages and birthweights |
| (Bora, Pritchard, Moor, Austin, & Woodward, 2011) | 212 (exposed=104) | 6 | Newzealand | Prospective | Preterm birth | ≤33 weeks gestation) | Child Behaviour Checklist (CBCL) | emotional and behavioral outcome | VPT children had odds of emotional, inattention/hyperactivity than their peers. |
| (Bos & Roze, 2011) | 160 | 6 to 12 | Netherlands | Prospective | Preterm birth | 24.0–31.6wk | Wechsler Intelligence Scale for Children, third edition, Gross Motor Function Classification System, Movement Assessment Battery for Children, | Motor function, intellectual ability, | Cases had abnormalities in motor and intellectual functioning. |
| (Brévaut-Malaty et al., 2010) | 283 | 6 to 10 | France | Prospective | Preterm birth | <32 weeks | Gross Motor Function Classification System, battery of tests for rapid evaluation, | motor function | 68% were normal  14% had minor disorders, and 18% had major disorders. |
| (Foulder-Hughes & Cooke, 2003) | 490 (exposed=280) | 7 to 8 | UK | Prospective | Preterm birth |  | MABC, COMPS, Developmental Test of Visual-Motor Integration,  Wechsler Intelligence Scale for Children, and Connors’ Teacher Rating Scale for attention-deficit–hyper- activity disorder |  | 23 have motor disability Cases had inattention and impulsivity and have a diagnosis of ADHD. |
| (Geldof et al., 2016) | 172 (exposed=105) | 5.5 | Netherlands | Prospective | Preterm birth | <32 weeks | Touwen examination, and Movement Assessment Battery for Children | Neurologic function | Visual and visual-motor functioning accounted for 9–11% of variance in MABC-2 Total, Manual Dexterity and Balance scores |
| (Geldof, van Wassenaer-Leemhuis, Dik, Kok, & Oosterlaan, 2015) | 106 | 5.5 | Netherlands | Prospective | Preterm birth | <32 weeks | Strengths and Difficulties Questionnaire, Children’s Social Behavior Questionnaire, | Cerebral visual impairment | Cases had lower IQ and visual problems |
| (Hadders-Algra, Huisjes, & Touwen, 1988) | 166 (exposed=80) | 6 | Netherlands | Prospective | Preterm birth | <37 weeks | Questinnaires, and neurologic classification based on Touwen | behavior and school achievement | 4% of the cases entered a special school |
| (Heinonen et al., 2015) | 919 (exposed=47) | 68.1 | Finland | Retrospective | Preterm birth | 34 weeks 0 days–36 weeks 6 days of gestation | Consortium to Establish a Registry for Alzheimer’s Disease Neuropsychological Battery, Mini–Mental State Examination | Neurocognitive outcomes | Cases had lower scores on word list recognition, and lower memory abilities than controls |
| (Hirvonen et al., 2017) | 1018256 (exposed=6329) | 7 | Finland | Retrospective | Preterm birth | <32+0 weeks | Bayley Scales of Infant Development, Wechsler Intelligence Scales for Children, parental interviews, observations and/or rating scales (e.g. Vineland Scales) | an intelligence quotient (IQ) at or below 70 Adaptive functioning |  |
| (Holmström & Larsson, 2008) | 199 | 10 | Sweden | Retrospective | Preterm birth | screened for retinopathy of prematurity (ROP) | visual acuity, strabismus, streopsis, cycloplegic refraction, fundus examination, contrast sensitivity, visual fields, color vision, questionnaire on school problems | visual function | 11 had normal visual acuity, 19 strabismus, and 2 subnormal contrast sensitivity alone. 3 had a combination of all 3 parameters, 8 had a combination of subnormal visual acuity and strabismus |
| (Jurgens-Van der Zee et al., 1979) | 1507 | 14 to 43 | Netherlands | Prospective | Preterm birth |  |  |  | 5.3% were neurologically abnormal |
| (Koç et al., 2016) | 90 | 6 to 8 | Turkey | Retrospective | Preterm birth | gestational age <32 weeks | Perinatal history, he Parent Evaluation of Developmental Status test, Pediatric Symptom Checklist, WISC-R | Intelligence, Neurological deficit, | 22% had cognitive problems and 1.7% with normal cognitive function also received special education. |
| (Kuban et al., 2016) | 889 | 10 | USA | Prospective | Preterm birth | gestational age <28 weeks | Differential Ability Scales–II, Oral and Written Language Scales, NEPSY-II, GMFCS, SCQ, ADOS-2 | Cognition Education, autism, gross motor, neurological status, communication function | Gross motor impairment remained even when children identified to have cerebral palsy at age 2 years were excluded |
| (Lacey & PhD, 1998) | 180 (exposed=153) | 6 | Australia | Prospective | Preterm birth |  | Touwen motor assessment | motor function | 7% were diagnosed as having CP |
| (Luu et al., 2009) | 486 (exposed=375) | 12 | USA | Prospective | Preterm birth |  | WISC-III, PPVT-R, CTOPP, CELF, VMI, TOWRE, GSRT, CBCL | Cognitive, language, memory, perceptual and motor functioning, behavior problems | 25% experienced difficulties in spoken and written language comprehension neurosensory impairment |
| (Talge et al., 2010) | 823 (exposed=473) | 6 | USA | Prospective | Preterm birth | 34 –36 weeks’ gestation | Wechsler Intelligence Scale for Children-Revised, CBCL, TRF | Cognitive function, behavior function | Cases had higher levels of internalizing and attention problems |
| (Van Baar, Van Wassenaer, Briët, Dekker, & Kok, 2005) | 157 | 5.5 | Netherlands | Retrospective | Preterm birth | <30 weeks | Touwen, Movement Assessment Battery for Children, Revised Amsterdam Children’s Intelligence Test, CBCL, TRF | Intellectual ability, motor, and behavior and emotional problems | 17% had a single disability, and 44% had multiple disabilities |
| (Dotinga et al., 2019) | 248 | 7 | Netherlands | Prospective | Preterm birth | birth at 32.0–35.9 weeks GA | CBCL | Emotional and behavioral problems | 22% had internalizing problems, 16.2% had externalizing problems; 19.4% had total problems |
| (Easson et al., 2019) | 128 | 11 to 19 | Netherlands | Retrospective | Preterm birth | ≤29 weeks of gestational age | SDQ, Leiter International Performance Scale-Revised, Movement Assessment Battery for Children-II | Cognition, behavior, and motor performance | 11.8% had cognition deficits, 50% had motor difficulties, 22.9% had behavioral problems |
| (Huddy, Johnson, & Hope, 2001) | 117 | 7 | USA | Retrospective | Preterm | 32–35 weeks gestation | SDQ | Behavior problems | 19% had abnormal hyperactivity |
| (van Schie et al., 2015) | 25 | 7.6 | Netherlands | Prospective | HIE | late decelerations or meconium-stained  amniotic fluid; umbilical cord pH < 7.10; respiratory insufficiency  at birth in need of mechanical ventilation; APGAR score  <5 at 5 minutes. | Movement Assessment Battery for Children-II, CBCL | Motor and behavior | 8 had cerebral palsy, 9 had motor impairment, 4 had behavior problems |
