# Supplementary material for: Long-term outcomes of survivors of neonatal insults: A systematic review and meta-analysis
Source: PLoS One. 2020 Apr 24;15(4):e0231947. doi: 10.1371/journal.pone.0231947 (PMC7182387; doi:10.1371/journal.pone.0231947)
Supplement: Attachment — Submitted filename: Response to Reviewers.docx [file pone.0231947.s006.docx]

Re: **PONE-D-19-22838 Long-term Outcomes of Survivors of Neonatal Insults: A Systematic Review and Meta-Analysis**

Dear Editor,

We thank you for your comments on the first version of our paper. Below you will find our responses to the reviewer’s comments and suggestions. With these revisions, we hope to have sufficiently addressed the reviewer’s concerns.

Thank you, also on behalf of my coauthors.

Kind regards,

Dorcas Magai, MSc.

**Review Comments to the Author**


**Methods:**

1) Is there a reason to choose the follow-up limit of 6 years?

- We considered children from six years of age, as this is the age where most children join elementary school, especially in low-and-middle-income countries. We have amended this part as follows:

*Criteria for eligible studies were: i) empirical studies conducted in humans; ii) the study participants had follow-up data coinciding with the age bracket at six years or older (we considered children from six years as this is the age where most children join elementary school, especially in low-and-middle-income countries) (page 5, line 63-66).*

**Data extraction and analysis**

2) Did you contact the authors for any missing data?

- We had 65 articles which could not be accessed online. Most of them were older than two decades (probably not archived online). Out of these, in 50 articles we were not able to find the contact details of the corresponding authors, in one article our email was not delivered (the author had probably left the institution and changed their email addresses). Nine authors did not respond to our emails even after three reminders. Five authors sent us full articles; however, these did not meet the inclusion criteria.

Additionally, there were 35 articles with a wide age range (e.g. 2 months to 10 years). We contacted authors of these studies to inquire whether they conducted a sub-group analysis for participants aged six years and above. However, we were not able to get this information from the contacted authors. We have edited figure 1 to capture this information.

**Results:**

3) Line 120: 94,978 this number includes control group as well?

- The 94,978 were survivors of neonatal insults. The control group is not included in the numbers, as indicated below.

*Overall, 94,978 survivors of NNI were included in the studies in this review* (page 7, line 122)*.*

4) Line 123-124: please mention the % of survivors in bracket, example NNJ (83.5%)

- We have amended this part as follows: *The most examined or studied NNI were HIE (15.4%), NNJ (15.4%), and preterm birth (42.3%)* (page 8, line 126).

5) Did you consider any NNJ or HIE as a neonatal insult? If yes, your sample size is diluted. It would help if you include high risk insults such as NNJ requiring intensive phototherapy or exchange transfusion and moderate to severe HIEs.

- The systematic review and meta-analysis aimed to examine the long-term impact of NNI globally. We considered all articles that provided a clear diagnosis of NNJ or HIE, and we did not limit the inclusion of these studies based on the severity of NNJ or HIE as we intended to determine if any of the children irrespective of the severity of their condition develop neurocognitive or mental health sequelae. Additionally, given the limited long-term outcome studies in these conditions, we included all the studies with a clear diagnosis of NNJ or HIE despite the severity of the condition. However, we have noted this as a limitation of our study as indicated below.

*Second, the inclusion of all children with a diagnosis of NNJ or HIE in the study without considering the severity levels might have diluted our sample* (page 15, line 243-245).

6) Similarly, a subgroup analysis of preterm birth by weight (ELBW, VLBW) will help.

- We have revised this part as follows: *We conducted a sub group analysis of 15 preterm birth studies with extremely low birth weight participants, and similar results were obtained* (page 8, line 138-139).

**Discussion**

7) Overall discussion is well written, however following points may need attention.
   Publication bias needs to be discussed.

- We have discussed the publication bias as follows:

*Lastly, the results of this study should be cautiously interpreted as we found publication bias indicated by the asymmetry forest plot, which may imply that studies with significant results were more likely to be published than those with non-significant results. The publication bias could also reflect the high heterogeneity in the studies included in this systematic review and meta-analysis (page 15, line 251-255).*

8) High heterogeneity is important. Random effect model may be useful, however a subgroup analysis of studies with lower heterogeneity is important (example I2 <60%)

- We thank the reviewer for this suggestion and acknowledge his recommendation for the subgroup analysis. However, the studies included in the meta-analysis in each condition are few and not optimal to conduct a subgroup analysis. We appreciate the reviewer’s recognition of the robustness of the random effect model in dealing with heterogeneity in studies.

9) No mention of any missing data and approach to address this important issue.

- In this systematic review and meta-analysis, we did not work with any primary data. We extracted secondary data from all the included studies, and we did not experience any missing data. Moreover, for the articles that we could not retrieve, their eligibility to be included in this study is doubtful since we are not sure whether they met the inclusion criteria for this systematic review and meta-analysis. We have corrected figure 1 to clarify that the 35 studies had a wide age range and not missing data as had previously been stated.
